# The Current and Future State of Vaccines, Antivirals and Gene Therapies Against Emerging Coronaviruses

**DOI:** 10.3389/fmicb.2020.00658

**Published:** 2020-04-24

**Authors:** Longping V. Tse, Rita M. Meganck, Rachel L. Graham, Ralph S. Baric

**Affiliations:** ^1^Department of Epidemiology, The University of North Carolina at Chapel Hill, Chapel Hill, NC, United States; ^2^Curriculum in Genetics and Molecular Biology, The University of North Carolina at Chapel Hill, Chapel Hill, NC, United States; ^3^Department of Microbiology and Immunology, The University of North Carolina at Chapel Hill, Chapel Hill, NC, United States

**Keywords:** coronavirus (CoV), vaccine, antivirals, adeno-associate virus, passive immunization strategy, MERS- and SARS-CoV, 2019 nCoV

## Abstract

Emerging coronaviruses (CoV) are constant global public health threats to society. Multiple ongoing clinical trials for vaccines and antivirals against CoVs showcase the availability of medical interventions to both prevent and treat the future emergence of highly pathogenic CoVs in human. However, given the diverse nature of CoVs and our close interactions with wild, domestic and companion animals, the next epidemic zoonotic CoV could resist the existing vaccines and antivirals developed, which are primarily focused on Severe Acute Respiratory Syndrome Coronavirus (SARS-CoV) and Middle East Respiratory Syndrome Coronavirus (MERS CoV). In late 2019, the novel CoV (SARS-CoV-2) emerged in Wuhan, China, causing global public health concern. In this review, we will summarize the key advancements of current vaccines and antivirals against SARS-CoV and MERS-CoV as well as discuss the challenge and opportunity in the current SARS-CoV-2 crisis. At the end, we advocate the development of a “plug-and-play” platform technologies that could allow quick manufacturing and administration of broad-spectrum countermeasures in an outbreak setting. We will discuss the potential of AAV-based gene therapy technology for *in vivo* therapeutic antibody delivery to combat SARS-CoV-2 outbreak and the future emergence of severe CoVs.

## Introduction

The zoonotic transmission and subsequent adaptation to humans of emerging RNA viruses is a global public health concern. In the 21st century alone, coronaviruses (CoV) have been responsible for two separate endemics, the severe acute respiratory syndrome (SARS) and Middle East Respiratory Syndrome (MERS) CoVs (de Wit et al., 2016). In late Dec 2019, a novel SARS-like CoV designated 2019 nCoV emerged in Wuhan China, causing > 60,000 cases and over 1350 deaths in an ongoing epidemic (Hui et al., 2020). Other highly pathogenic threat viruses that have emerged in the 21st century include influenza viruses, Ebola viruses, flaviviruses and paramyxoviruses (Mackenzie and Jeggo, 2013). The high mutation and recombination rate of RNA viruses drives the emergence of new viral strains that can rapidly adapt to new and changing ecologies (Drake and Holland, 1999; Lauring and Andino, 2010). Furthermore, industrialization, globalization and traditional cultural habits potentiate the likelihood of zoonotic transmission and facilitate the spread of viruses in the human population. While new outbreaks from emergent viruses are inevitable, scientists, epidemiologists, and the health care industry are racing to develop new technologies to better predict and minimize the impact of an outbreak by employing global viral surveillance programs and developing vaccines and antivirals (Lipkin and Firth, 2013). A major challenge of vaccine and antiviral development is the elusive nature of the emerging viruses, which oftentimes emerge from highly heterogeneous populations of virus strains that circulate in animal reservoirs (Lauring and Andino, 2010). Therefore, to prepare for future outbreaks, vaccines and antivirals will need to be both potent and broadly effective against multiple potential emerging viruses within and across virus families. Additionally, in order to control and prevent viral spread, treatments must be readily available to affected populations and have a fast response time. In this review, we will focus our discussion on the challenges, as well as current development, of vaccines and antivirals for SARS-CoV and MERS-CoV. At the end, we will also discuss the potential use of AAV-based gene therapy as a quick response to prevent and treat emerging viral infections in the current SARS-CoV-2 and future outbreak situations.

### Endemic and Emerging Coronaviruses

**FIGURE 1:**
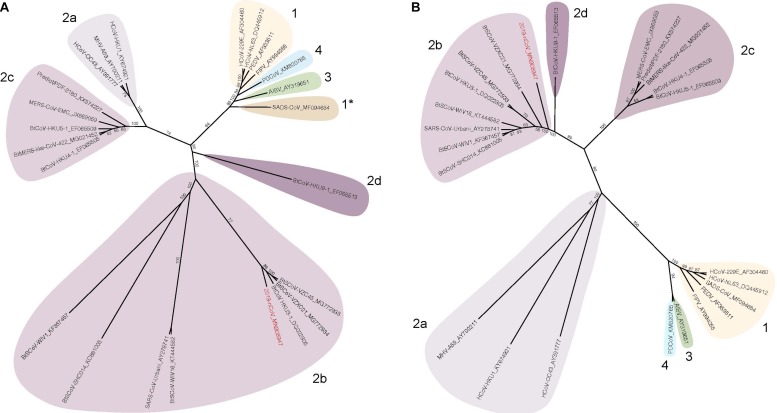
Spike and nsp12 phylogeny of representative coronaviruses. The Spike **(A)** and nsp12 **(B)** protein sequences of selected coronaviruses were aligned and phylogenetically compared. Coronavirus genera are grouped by classic subgroup designations (1, 2a-d, 3, and 4). In the Spike tree in **(A)**, SADS-CoV is designated as 1* because of its distinctive grouping compared with more conserved proteins (e.g., nsp12, see **(B)**). Branches in each tree are labeled with consensus support values (in %). Sequences were aligned using free end gaps with the Blosum62 cost matrix, and the tree was constructed using the neighbor-joining method based on the multiple sequence alignment in Geneious Prime. Numbers following the underscores in each sequence correspond to the GenBank Accession number. The SARS-CoV-2 is highlighted in red. The radial phylogram was exported from Geneious and then rendered for publication using Adobe Illustrator CC 2020.

Coronaviruses are a diverse group of positive-stranded RNA viruses which infect a wide range of animals from birds to mammals, causing a variety of diseases (Perlman and Netland, 2009; Woo et al., 2009; Peck et al., 2015). Based on sequence identity of the spike protein or the non-structural proteins (nsp), CoVs are classified into four different sub-groups, *alphacoronaviruses*, *betacoronaviruses*, *gammacoronaviruses*, and *deltacoronaviruses* (Figure 1). Human coronaviruses (hCoVs), such as 229E, OC43, NL-63 and HKU-1 are highly transmissible respiratory viruses which are responsible for around 10-20% of common cold cases annually (McIntosh et al., 1970; Cabeça et al., 2013). HCoV-related illness is often self-limited in immune competent individuals but may cause more severe upper and lower respiratory tract infections in the young and elderly population (Woo et al., 2005; Lau et al., 2006). In addition, highly pathogenic CoVs may emerge through zoonotic reservoirs. In the past two decades, SARS-CoV and MERS-CoV emerged from bats and spread to humans through intermediate hosts including civet cats and camels, respectively (Raj et al., 2014). SARS-CoV and MERS-CoV belong to the sub-groups 2b and 2c of the *Betacoronavirus* genus (Peck et al., 2015). The latest CoV outbreak is the SARS-CoV-2, a *Betacoronavirus* 2b which emerged from bats and spread to humans (Lu et al., 2020). The mortality rate of these viruses range from 10 to 40% but can exceed 50% in the elderly (Min et al., 2004; Li et al., 2005; Bolles et al., 2011b; Raj et al., 2014; Sharif-Yakan and Kanj, 2014; World Health Organization [WHO], 2018). The unusually high mortality rate is linked to disease progression leading to acute respiratory distress syndrome (ARDS) which causes hypoxemia, pulmonary edema, and infiltration of inflammatory immune cells in the lung (Cabeça et al., 2013; Gralinski and Baric, 2015). If unresolved, the diseases progress to late phase ARDS, leading to end-stage lung disease and death (Ding et al., 2003). Currently, no vaccines or antiviral drugs are approved to prevent or treat severe CoV infection.

### The Challenge for Vaccine Development

The CoV *S*-protein is the major envelope glycoprotein and the main determinant of protective immunity. The *S*-protein is composed of two principle subunits, S1 and S2; S1 governs receptor binding and S2 is responsible for membrane fusion (Li, 2016). Similar to other class I fusion proteins, *S*-protein undergoes a major conformational change between pre-fusion and post-fusion which also presents different antigenic epitopes. While able to bind to both conformations, Abs targeting the post-fusion form are not necessary neutralizing; in contrast, Abs targeting the pre-fusion form of the *S*-protein correlate better with neutralization. In particular, vaccines and neutralizing antibodies (nAbs) which target the receptor binding domain (RBD) of the *S*-protein can effectively neutralize the virus (Zhu et al., 2007; Tang et al., 2014). However, due to high selective pressure and tropism determinants, *S*-protein is the most diverse region of CoV. For instance, the *S*-proteins from SARS-CoV and MERS-CoV share only 44% sequence identity (Kandeel, 2018). The majority of differences between *S*-proteins is in the S1 region, which is further separated into the N-terminal Domain (NTD) and RBD. The diverse nature of the RBD between SARS-CoV and MERS-CoV is reflected in the use of different entry receptors, either angiotensin I converting enzyme 2 (ACE2) or dipeptidyl peptidase 4 (DPP4), respectively (Li et al., 2003; Raj et al., 2013). The diversity of *S*-protein also renders vaccines and nAbs unlikely to be cross-protective between existing and emerging CoVs. Surveillance and experimental data have identified multiple animal SARS- and MERS-like CoVs that have significant diversity in *S*-protein and are able to replicate in human cells without adaptation (Menachery et al., 2015, 2016; Luo et al., 2018). Once transmitted to human population, such variation between *S*-protein between the previous and the new emerging CoVs will pose a major challenge on the progress of vaccine development.

## Criteria for Generating Effective Vaccine for CoVs

### Vaccine Criteria for SARS and MERS-CoV Viruses

The surface glycoproteins are the main target for vaccine development. In CoV infection, Abs against *S*-protein were shown to be protective in multiple animal studies. Furthermore, in a passive immunization study in camel, the nAb level is directly correlated to lung pathology and survival (Zhao et al., 2015). As such, one of the main goals for CoV vaccines in humans is the ability to elicit a strong humoral immune response against the *S*-protein. Particularly, the pre-fusion form of the *S*-protein is an attractive target for Abs to confer protective immunity. In order to “lock” the *S*-protein in its antigenic optimal pre-fusion form, two mutations, V1060P and L1061P, were introduced into the MERS-CoV *S*-protein (Pallesen et al., 2017). The resulting MERS S-2P is able to elicit both RBD and non-RBD binding nAbs (Pallesen et al., 2017). The same strategy was also shown to work in the SARS-CoV-2 *S*-protein (Wrapp et al., 2020). Other structural proteins such as E and M and the nsp N also contribute to viral protection and clearance (Channappanavar et al., 2014; Zhao et al., 2016; Deng et al., 2018). Similarly to other respiratory viruses such as influenza, mucosal IgA plays a major role in disease protection and has a synergistic effect with IgG (Belshe et al., 2000; Plotkin, 2010). In order to elicit a strong mucosal IgA immunity, the route of vaccine administration is important. Studies have shown that intranasal inoculation of a recombinant RBD vaccine can elicit greater mucosal IgA production than intramuscular or subcutaneous injection (Ma et al., 2014a). However, the duration of mucosal antibodies is typically shorter lived than systemic IgG responses and the longest longitudinal study of MERS-CoV IgA responses ended after 6 months (Hapfelmeier et al., 2010; Ma et al., 2014a). In comparison, in a natural infection case study, SARS-targeting systemic memory B cells were present up to 6 years for SARS-CoV (Oh et al., 2012; Tang et al., 2019); and up to 34 months post infection for MERS-CoV (Payne et al., 2016). Another important consideration for SARS-CoV and MERS-CoV vaccination is the T cell response against the virus, specifically the N proteins, which is important for viral clearance (Zhao et al., 2010; Channappanavar et al., 2014). A study has shown that adoptive transfer of viral specific T-cells to SCID mice enhances survival and reduces lung titer after SARS-CoV infection (Zhao et al., 2010). Moreover, intranasal vaccination of N protein using the VEEV replicon system elicits CD4+ memory T-cells responses in the airway. Upon challenge, the airway CD4+ memory T cells secrete IFN-γ, which subsequently enhances the innate immune response as well as coordinates the CD8+ T cell priming and migration which protects mice from lethal disease, but not weight loss or virus titers under carefully controlled conditions (Zhao et al., 2016). Interestingly, Rag−/− mice are able to clear SARS-CoV infection, suggesting innate immunity is sufficient for viral clearance (Zhao et al., 2010). However, the mechanism of this viral clearance is still unknown. Another important aspect of emerging CoV vaccine is the breadth of protection. As mentioned previously, the antigenic variation in the *S*-protein between CoVs limits the breadth of cross protection against multiple emerging CoVs, and is especially true for *S*-protein only vaccines (Wang et al., 2018, 2019).

Learning from natural infection, MERS-CoV specific CD4+ and CD8+ T cells are detected in PBMCs in MERS-CoV infected survivors (Zhao et al., 2017). Therefore, a balance of B cell and T cell responses is generally considered the gold standard to prevent and resolve MERS-CoV and SARS-CoV infection. Multiple strategies have been developed to elicit long lasting B and T cell responses for SARS-CoV and MERS-CoV. These include the traditional live attenuated, inactivated, and subunit vaccines, and newer development of nanoparticles, vectorized vaccines, and RNA/DNA vaccines. In this review, we have selected only the vaccine studies that have an *in vivo* challenge model and have summarized the different parameters, including vaccine components, dosage, challenge conditions, animal models and the study outcome in Table 1. We will also discuss each type of vaccine strategy and focus on the finished clinical trial targeting SARS-CoV and the 3 ongoing clinical trials targeting MERS-CoV using DNA (Martin et al., 2008; Modjarrad et al., 2019) and vectorized vaccines. Other comprehensive reviews on CoV vaccine development can be found elsewhere (Zhang et al., 2014; Du and Jiang, 2015; Perlman and Vijay, 2016; Schindewolf and Menachery, 2019; Yong et al., 2019).

**TABLE 1:** Summary of SARS-CoV and MERS-CoV vaccines studies

**SARS-CoV Vaccines**	**Antigens**	**Vaccine formulations**	**Dose, Time of vaccination**	**Animal models**	**Challenge dose. Virus strains, Time after last vaccination, Route**	**Results**	**References**
Whole Inactivated Vaccine	Inactivated	SARS-CoV FRA (*b*-propiolactone) + MF59	5 μg at 0, 2, and 4 weeks, SC	BALB/c	10^4^ TCID50 SARS-CoV Urbani, IN	Protection, no virus detected in Lung and Nasal Turbinates	Stadler et al., 2005 EID
	Inactivated	SARS-CoV Tor2 (*b*-propiolactone) + Alum	50 μg at 0 and 4 weeks, SC	6 week old 129S6/SvEv	10^6^ pfu SARS-CoV Tor2 at week 3, IN	Protection	See et al., 2006 JGV
	Double Inactive	SARS-CoV (Utah) formaldehyde and UV inactivation + Al(OH)3	0.08 – 0.2 μg at week 0 and 2, SC	6 – 8 weeks CD1 mice	10^5^ TCID50 SARS-CoV Utah at week 3 or 11, IN	Protection, no virus detected in lung and trachea	Spruth et al., 2006 Vaccines
	Inactivated	SARS-CoV Tor2 (*b*-propiolactone) + Alum	50 μg at 0 and 4 weeks, SC	8 – 10 month old ferret	10^6^ pfu SARS-CoV Tor2 at week 3, IN	Weak protection, slight reduction of viral titer in lung, BAL and nasal wash, slight improvement in lung pathology	See et al., 2008 JGV
	Inactivated	SARS-CoV Urbina (*b*-propiolactone) + AS01B and AS03A	0.15 – 1.5 μg at week 0 and 3, IM	BALB/c	10^5^ TCID50 SARS-CoV Urbani at week 3, IN	Protection, no virus detected in lung	Roberts et al., 2010 Viral Immunology
	Inactivated	SARS-CoV Urbina (*b*-propiolactone) + AS01B and AS03A	2.0 μg at week 0 and 3, IM	4 – 8 week old Golden syrian hamsters	10^3^ TCID50 SARS-CoV Urbani at week 4 or 18, IN	Partial protection, reduce viral titer in lung	Roberts et al., 2010 Viral Immunology
	Double Inactive	SARS-CoV MA15 formalin and UV inactivated + Alum	0.2 μg at week 0 and 3–4 week, footpad	6 – 8 weeks old BALB/cAnNHsd	10^5^ PFU SARS-CoV MA15, SZ61and GD03 at week 5, IN	Partial protection, reduce viral titer in lung and good protection from lethal challenge (MA15)	Bolles et al., 2011a JVI
	Double Inactive	SARS-CoV MA15 formalin and UV inactivated + Alum	0.2 μg at week 0 and 3–4 week, footpad	12 – 14 months old BALB/cAnNHsd	10^5^ PFU SARS-CoV MA15, SZ61and GD03 at week 5, IN	Weak protection, small drop in viral titer in lung, weak protection from leathal challenge (MA15)	Bolles et al., 2011a JVI
LAV	SARS-CoV-ΔE (Urbani)	SARS-CoV-ΔE (Urbani)	10^3^ TCID50 at 0 week, IN	7 week old Golden syrian hamsters	10^3^ TCID50 SARS-CoV Urbani and GD03 at week 4, IN	Protection, no virus detected in lung	Lamirande et al., 2008 JVI
	SARS-C0V ExoN (MA15)	SARS-CoV-ExoN (MA15)	10^2.5^ or 10^4^ PFU at week	12 months old BALB/c	10^2.5^ TCID50 SARS-CoV MA15 at week 4, IN	Complete survival from lethal challenge, no virus detected in lung	Graham et al., 2012 Nat Med
	SARS-CoV ΔNSP16	SARS-CoV Δ2′-*O*-Methyltransferase	10^2^ PFU at week 0, IN	10 weeks old BALB/c and C57BL/6	10^5^ PFU SARS-CoV MA15 at week 4, IN	Complete survival from lethal challenge, no viral titer performed	Menachery et al., 2014 JVI
	SARS-CoV ΔNSP16/ExoN	SARS-CoV Δ2′-*O*-Methyltransferase and ExoN mutation	10^2^ PFU at week 0, IN	12 months old BALB/c	10^5^ PFU SARS-CoV MA15 at week 4, IN	Complete survival from lethal challenge, no virus detected in lung	Menachery et al., 2018 JVI
Subunit	subunit nS Sf9	SARS-CoV S (14-762) Urbani + QS21	10 μg at week 0, 4, and 8, SC	6 weeks old BALB/c	10^5^ TCID50 SARS-CoV (Urbani) at week 4, IN	Protection, reduce viral titer in lung and nasal turbinates	Bisht et al., 2005 Virology
	Ectodomain Sf9	SARS-CoV S ectodomain (Urbani) + Protollin	10 or 30 μg at week 0, 2 and 5, IN	∼ 1 year old BALB/c	5x10^4^ TCID50 SARS-CoV Urbani at week 1, IN	Protection, reduce viral titer in lung	Hu et al., 2007 Vaccines
	Subunit RBD219 CHO	SARS-CoV RBD (318-536)Tor2 + Freund	20 μg at week 0 and 10 μg at week 3 and 6, SC	4 – 6 weeks old BALB/c	5x10^5^ TCID50 SARS-CoV GZ50 at 10 day, IN	Protection, no virus detected in lung	Du et al., 2010 viral immunology
Subunit Trimer	Spike trimer	SARS-CoV RBD Spike Trimer Urbani + Alum	50 μg at week 0, 3, and 6, SC	5 weeks old Golden syrian hamsters	10^3^ TCID50 SARS-CoV Urbani at week 2, IN	Protection, no virus detected in lung and reduce pneumonitis	Kam et al., 2007 Vaccines
VLP	MHV-S VLP	SARS-CoV S in MHV + Alum	2 μg at 0 and 4 weeks	6 – 8 week old BALB/c	10^6^ TCID50 SARS-CoV at week 8, IN	Protect, no virus detected in Lung	Lokugamage et al., 2008 Vaccine
	Flu M1-S	SARS-CoV S Urbani in influenza M1 + Al(OH)3	0.8 or 4 μg at week 0 and 3, IM	6 – 8 weeks old BALB/c	2xLD50 mouse adapted SARS-CoV V2163 at week 3, IN	Complete survival from lethal challenge, no virus detected in lung	Liu et al., 2011 Vaccines
DNA	Plasmid *S*-Ectodomain	Plasmid expressing SARS-CoV S ΔCD or ectodomain (Urbani)	25 μg at week 0, 3, and 6, IM	6 – 8 week old BALB/c	10^4^ TCID50 SARS-CoV Urbani, at Day 30, IN	Protection, reduce viral titer in lung and nasal turbinates	Yang et al., 2004 Nature
Vector	BHPIV3 SARS-S	SARS-CoV S or SME Urbani in parainfluenza virus type 3 vector (BHPIV3)	10^6^ TCID50 at 0 week, IN	Golden syrian hamsters	10^3^ TCID50 SARS-CoV Urbani at week 4, IN	Protection, no virus detected in lung	Buchholz et al., 2004 PNAS
	BHPIV3 SARS-S	SARS-CoV S Urbani in parainfluenza virus type 3 vector (BHPIV3)	10^6^ TCID50 each at 0 week, IN and IT	African green monkeys	“A large dose of SARS-CoV” at week 4, IN	Protection, no virus detected in lung	Bukreyev et al., 2004 Lancet
	MVA-SARS-S	SARS-CoV S Urbani in MVA	10^7^ at 0 and 4 week, IN	BALB/c	10^4^ TCID50 SARS-CoV Urbani, at week 8, IN	Protection, reduce viral titer in lung and nasal turbinates	Bisht et al., 2004 PNAS
	MVA-SARS-S	SARS-CoV S Urbani in MVA	10^7^ at 0 and 4 week, IM	BALB/c	10^4^ TCID50 SARS-CoV Urbani, at week 8, IN	Protection, reduce viral titer in lung and nasal turbinates	Bisht et al., 2004 PNAS
	MVA-SARS-S	SARS-CoV S and N Tor2 in MVA	10^8^ pfu at 0 and 5 × 10^7^ at 2 week, IP and SC	Ferret	10^6^ pfu SARS-CoV Tor2 at week 4, IN	No protection according to viral RNA in lung, Ab induction	Czub et al., 2005 Vaccines
	VSV-S	VSV-SARS-S Urbani	1.4 × 10^4^ pfu at week 0, IN	BALB/c	10^4^ TCID50 SARS-CoV Urbani at month 1 and 4, IN	Protection, no virus detected in lung and nasal turbinates	Kapadia et al., 2005 Virology
	hAd5 (N+S)	Human Adenovirus 5 with S + Ad5 N gene	3 × 10^8^ pfu each at 0 and 4 weeks, IM	6 week old 129S6/SvEv	10^6^ pfu SARS-CoV Tor2 at week 3, IN	No protection despite strong IgG1, IgG2a Ab induction and high IFN-g secretion	See et al., 2006 JGV
	hAd5 (N+S)	Human Adenovirus 5 with S + Ad5 N gene	3 × 10^8^ pfu each at 0 and 4 weeks, IN	6 week old 129S6/SvEv	10^6^ pfu SARS-CoV Tor2 at week 3, IN	Partial protection, Induction of IgA	See et al., 2006 JGV

**MERS-CoV Vaccines**	**Antigens**	**Vaccine formulations**	**Dose, Time of vaccination**	**Animal models**	**Challenge dose. Virus strains, Time after last vaccination, Route**	**Results**	**References**

	VEEV-S	SARS-CoV GD03 S in VEEV	10^6^ IU at 0 week, boost 3 – 5 weeks, footpad	4 – 7 weeks old	10^5^ pfu SARS-CoV GD03 and Urbani at week 7 – 8 and week 54, IN	Protection, no virus detected in lung	Deming et al., 2006 PlOS Med
	VEEV-S	SARS-CoV GD03 S in VEEV	10^6^ IU at 0 week, boost 3 - 5 week, footpad	> 26 week old	10^5^ pfu SARS-CoV GD03 and Urbani at week 7 -8 and week 54, IN	No protection	Deming et al., 2006 PlOS Med
	hAd5-S + AdC7-S	Human Adenovirus 5 with S + Chimpanzee AdC7 with S Tor2	5 × 10^11^ VP/kg at 0 and 1 month, IM	18 – 20 weeks old ferret	10^6^ pfu SARS-CoV Tor2 at not specify, IN	Protection, reduce viral titer in lung and nasal turbinates	Kobinger et al., 2007 Vaccines
	hAd5 (N+S)	Human Adenovirus 5 with S + Ad5 N gene	1 × 10^9^ pfu each at 0 and 4 weeks, IM	8 - 10 month old ferret	10^6^ pfu SARS-CoV Tor2 at week 3, IN	Inconclusive, control hAd5 shows non-specific protection	See et al., 2008 JGV
	hAd5 (N+S)	Human Adenovirus 5 with S + Ad5 N gene	1 × 10^9^ pfu each at 0 and 4 weeks, IN	8 – 10 months old ferret	10^6^ pfu SARS-CoV Tor2 at week 3, IN	Inconclusive, control hAd5 shows non-specific protection	See et al., 2008 JGV
Whole Inactivaed Vaccine	Inactivated	MERS-CoV (γ-ray) + Alum or MF59	10^6^ TCID50 at week 0 and 3, IM	hCD26/DPP4 transgenic mice	10^3^ TCID50 (100xLD50) MERS-CoV at week 3, IN	Protection from virus replication, no virus detected in lung, increase lung pathology	Agrawal et al., 2016 Human vaccine and immuno therapy
	Inactivated	MERS-CoV EMC/2012 (formaldehyde) + Alum and CpG	1 μg at week 0, 4 and 8, IM	14 – 16 week old BALB/c transduced with hDPP4 by Ad5	10^5^ PFU MERS-CoV (EMC/2012) at week 2, IN	Protection, no virus detected in lung	Deng et al., 2018 Emerging Microbes and infection
LAV	MERSS-CoV ΔNSP16	MERS-CoV Δ2′-*O*-Methyltransferase EMC MA1	10^6^ PFU at week 0, IN	10 - 20 week old C57BL/6 288-330+/+	10^6^ PFU MERS-CoV EMC MA1 at week 4, IN	Complete survival from lethal challenge, reduce viral titer in lung	Menachery et al., 2017 msphere
Subunit	RBD	MERS-CoV RBD S367-606 (EMC/2012) + alum	200 μg at week 0 and 100 μg at week 8 and 25, IM	Rhesus Macaque	6.5x10^7^ TCID50 MERS-CoV (EMC/2012) at week 2, IN	Partial protection, reduce viral titer in lung and trachea	Lan et al., 2015 EBioMedicine
	NTD	MERS-CoV NTD S18-353 (EMC/2012) + alum and CpG	5 or 1 μg at week 0, 4 and 8, IM	16 – 18 weeks old BALB/c transduced with hDPP4 by Ad5	10^5^ PFU MERS-CoV (EMC/2012) at week 2, IN	Partial protection, reduce lung and trachea pathologies, no viral titer information	Jiaming et al., 2017 Vaccines
	S ectodomain	MERS-CoV EMC/2012 S ectodomain + Alum and CpG	1 μg at week 0, 4 and 8, IM	14 – 16 week old BALB/c transduced with hDPP4 by Ad5	10^5^ PFU MERS-CoV (EMC/2012) at week 2, IN	Protection, reduction in lung viral titer	Deng et al., 2018 Emerging Microbes and infection
	RBD-Fc	MERS-CoV RBD S377-588 Fc (EMC/2012) + MF59	10 μg at week 0, 3, and 6, SC	4 – 6 weeks old BALB/c transduced with hDPP4 by Ad5	10^5^ PFU MERS-CoV (EMC/2012) at week 2, IN	Protection, no virus detected in lung	Zhang et al., 2016 Cellular and molecular immunology
	RBD-Fc	MERS-CoV RBD S377-588 Fc (EMC/2012) + Addavax	10 μg at week 0 and 4, IM	hCD26/DPP4 transgenic mice	10^3^ TCID50 (100xLD50) MERS-CoV (EMC/2012) at week 4, IN	Complete survival from lethal challenge, no virus detected in lung	Nyon et al., 2018 Vaccines
	S1	MERS-CoV ENgland1 S1 + Advax HCXL or Sigma oil-in-water emulsion	400 μg at week 0, 4 and 15, IM	dromedary camel	10^7^ TCID50 MERS-CoV (EMC/2012) at ∼week 4, IN	Protection, reduce viral titer in lung and nasal turbinate	Adney et al., 2019 Virus
	S1	MERS-CoV ENgland1 S1 + Advax HCXL or Sigma oil-in-water emulsion	400 μg at week 0, 4, and 15, IM	Alpaca	10^7^ TCID50 MERS-CoV (EMC/2012) at ∼week 4, IN	Protection, no virus detected in lung, nasal turbinate and nasal swabs	Adney et al., 2019 Virus
Subunit Trimer	RBD Trimer	MERS-CoV RBD-Fd (Trimer) + alum	5 μg at week 0 and 4, IM	hCD26/DPP4 transgenic mice	10^4^ TCID50 MERS-CoV (EMC/2012) at week 12, IN	Complete survival from lethal challenge, no viral titer performed	Tai et al., 2016 Virology
VLP	S nanoparticles	MERS-CoV Jordan S nanoparticle + Matrix M1	10 μg at week 0 and 3, IM	15 – 17 weeks old BALB/c transduced with hDPP4 by Ad5	2.5 × 10^3^ PFU MERS-CoV (Jordan) at week 4, IN	Protection from chalenge, no virus detected in lung	Coleman et al., 2017 Vaccines
	BNSP333-S	MERS-CoV S1 Jordan fused with rabies virus G protein VLP	10 μg at week 0, 1, and 3, IM	15 – 17 weeks old BALB/c transduced with hDPP4 by Ad5	2.5 × 10^3^ PFU MERS-CoV (Jordan) at week 4, IN	Protection, no virus detected in lung	Wirblich et al., 2017 JVI
DNA	Plasmid S-protein	Plasmid expressing MERS-CoV S1 (Al-Hasa_1_2013)	0.5 or 2 mg at week 0, 3 and 6, IM	Rhesus macaques	7 × 10^6^ TCID50 MERS-CoV (EMC/2012) at week 5, IT, IN, oral and ocular	Protection, reduce viral titer in lung, reduce clinical pathology	Muthumani et al., 2015 STM
	Plasmid S1	Plasmid expressing MERS-CoV S1 (Al-Hasa_15_2013)	100 μg at week 0, 3 and 6, IM	14 - 16 week old BALB/c transduced with hDPP4 by Ad5	10^5^ PFU MERS-CoV (EMC/2012) at Day 18, IN	Protection, reduce viral titer in lung	Chi et al., 2017 Vaccines
Vector	MVvca2 S or soluble S	Recombinant measles virus with MERS-CoV (EMC/2012) S or soluble S	10^5^ TCID50 at week 0 and 4, IP	6 – 12 week old IFNAR−/− CD46Ge transduced with hDPP4 by Ad5	7 × 10^4^ TCID50 MERS-CoV (EMC/2012) at week 6, IN	Protection, reduce viral titer in lung, reduce lung pathology	Malczyk et al., 2015 JVI
	ChAdOx1 MERS	ChAdOx1 with MERS-CoV *S*-protein (EMC/2012)	10^8^ IU at week 0, IN or IM	hCD26/DPP4 transgenic mice	10^4^ TCID50 MERS-CoV (EMC/2012) at week 4, IN	Complete survival from lethal challenge, no virus detected in lung, reduce lung pathology	Munster et al., 2017 npj
	MVA-MERS-S	MVA with MERS-CoV *S*-protein	10^6^, 10^7^, or 10^8^ PFU at week 0, IM or SC	14 – 16 week old BALB/c transduced with hDPP4 by Ad5	7 × 10^4^ TCID50 MERS-CoV (EMC/2012) at week 6, IN	Protection, reduce viral RNA genome in lung, reduce lung pathology	Volz et al., 2015 JVI
	rAd5-S1/F/CD40L	hAd5 with MERS-CoV S1 trimer fused with CD40L	10^9^ PFU at week 0 and 4, IM	hCD26/DPP4 transgenic mice	10^3^ TCID50 (100xLD50) MERS-CoV (EMC/2012) at week 4, IN	Complete survival from lethal challenge, no virus detected in lung, reduce lung pathology	Hashem et al., 2019 JID

*Selection criteria: Only animal studies with a challenge model are included. LAV, live attenuated vaccine; VLP, virus like particle; RBD, receptor binding domain; IM, intramuscular; IN, intranasal; SC, subcutaneous; IP, intraperitoneal.*

### Current Vaccine Strategies

Inactivated vaccines are the quickest option for vaccine development in an outbreak situation. Multiple chemical and physical methods have been applied singly or in combination to inactivate CoVs, including β-propiolactone, formalin, formaldehyde and UV. While multiple studies have shown the efficacy of inactivated vaccines in hamster, ferret and multiple mouse challenge models (Stadler et al., 2005; See et al., 2006, 2008; Spruth et al., 2006; Roberts et al., 2010), one study suggested a potential vaccine enhanced pathologies (Bolles et al., 2011a). In this study, double inactivation (DIV) of SARS-CoV using formalin and UV+alum elicits a Th2 skewed response and is only partially protective to young mice (6–8 weeks-old) and not protective to experimentally aged mice (12–14 weeks-old) (Bolles et al., 2011a). Furthermore, upon challenge, DIV vaccinated mice show increased infiltration of eosinophils, neutrophils and other inflammatory cell populations in the lung, likely due to the N specific immune response (Bolles et al., 2011a). Further studies have suggested that by replacing alum with TLR agonists such as Poly I:C, Poly U or LPS as adjuvants, the skewed Th2 responses can be alleviated and may reduce the infiltration of eosinophils in the lung (Iwata-Yoshikawa et al., 2014).

Gamma-ray inactivated whole MERS-CoV (WIV) with alum or M59 also suffers from the Th2 skewed immune response and pulmonary eosinophilia upon challenge, suggesting a potential risk of using inactive virus for CoV vaccination (Agrawal et al., 2016). Interestingly, formaldehyde inactivated MERS-CoV co-administered with alum and CpG shows a more balanced Th1/Th2 response and is able to protect mice from a challenge model (Ad5 transduced hDPP4) with reduction in lung viral titer and improved lung pathology. There is no observable vaccine-induced lung pathology or infiltration of eosinophils upon challenge (Deng et al., 2018). The difference in vaccine outcomes indicates an incomplete understanding of the effect of adjuvants on the inactivated SARS and MERS-CoV vaccines. In respiratory syncytial virus (RSV) vaccines, formalin inactivated vaccines presents the post-fusion form predominately and fail to elicit protective immune responses. Whether the same phenomenon exists in inactivated CoV is still unknown, although it could explain the discrepancy between reports. The inconsistent results from different vaccine models also underscores the host genetic elements affecting vaccine outcome. Nevertheless, inactivated vaccine is still one of the most straightforward methods for vaccine development and has the quickest response time in an outbreak situation.

Live attenuated viral vaccines are the closest mimic of natural infection and generally elicit strong B and T cell responses (Zhao et al., 2017). Multiple strategies have been used to genetically attenuate SARS-CoV and MERS-CoV by either deleting or mutating structural, non-structural or accessory proteins. Intranasal immunization of a SARS-CoV lacking E protein (rSARS-CoV-ΔE) has shown complete protection from pulmonary replication in a Golden Syrian hamster model (Lamirande et al., 2008). A similar virus has been generated in the MERS backbone, creating a conditional mutant that requires trans expression of E for productive replication (Almazán et al., 2013). Mutations of the DEDD motif of the 3′ to 5′ exonuclease (ExoN-nsp14) “proof-reading protein” on a mouse-adapted SARS-CoV attenuated the virus both *in vitro* and *in vivo*. A single intranasal immunization is able to elicit strong nAbs (>6-fold protective titers) and completely protect against lethal challenges in an aged mouse model (12 months-old BALB/c) (Graham et al., 2012). Mutations in the nsp 16 (NSP16), a 2′*O*-methyltransferase, in both SARS-CoV and MERS-CoV have also been shown to attenuate the viruses and to protect BALB/c and CRISPR-Cas humanized DPP4-288-330 mice from lethal challenge (Menachery et al., 2014, 2017, 2018). Moreover, these attenuation strategies can be multiplexed, leading to highly stable live attenuated vaccines with limited capability to undergo recombination and reversion repair (Graham et al., 2018; Menachery et al., 2018). Although the live attenuated vaccine is effective in small animal models, there remain safety concerns about potential revertants and recombination with natural CoVs which hinders their usage in the clinical setting. Furthermore, live attenuated vaccines often require greater time for development and safety testing which lessens utility in an outbreak situation.

Protein-based subunit vaccines are considered the safest format of vaccine. However, the low immunogenicity of subunit vaccines dictates a heavily reliance on adjuvants. Different forms of the *S*-protein, including the S1 RBD, RBD-Fc (RBD with human IgG Fc fusion), and N-terminal domain (NTD), have demonstrated various degrees of nAb responses and protection in multiple animal models including non-human primates (NHP) (Lan et al., 2015; Zhang et al., 2016; Jiaming et al., 2017; Wang et al., 2017; Deng et al., 2018; Nyon et al., 2018; Adney et al., 2019). For instance, the SARS-CoV S1 subunit vaccine produced from sf9 cells and with the adjuvant saponin or protollin are able to reduce lung viral titer in young or aged mice after challenge, respectively (Bisht et al., 2005; Hu et al., 2007). An RBD subunit of SARS-CoV produced from Chinese hamster ovarian (CHO) cells with Freund’s adjuvant is able to protect young BALB/c mice from infection (Du et al., 2010). MERS-CoV-S1 with adjuvants MF59 or Advax HCXL is able to protect alpacas and dromedary camels against MERS-CoV challenge (Adney et al., 2019). Adjuvant selection can affect the vaccine outcome, and combinations of adjuvants can have synergistic effects on the strength of the response. For instance, rRBD with adjuvants alum and CpG ODN together elicits a stronger humoral and cellular T cell response (Lan et al., 2014). Additionally, rNTD with alum is able to reduce lung pathology in a non-lethal MERS-CoV challenge (Jiaming et al., 2017). Instead of adjuvants alone, immune enhancers such as an Fc fragment, which increases the protein half-life when fused with the RBD, can also elicit a stronger IgG nAb and cellular immune response in multiple experimental animals (Du et al., 2013; Ma et al., 2014b; Tang et al., 2015; Nyon et al., 2018). RBD-Fc fusion subunit vaccine is able to protect a lethal challenge of MERS-CoV in adenovirus transduced hCD26/DPP4 mice (Zhang et al., 2016; Wang et al., 2017). While a large amount of work concerning the subunit vaccines has been done in conjunction with different adjuvants, the effect of each adjuvant is not well understood and multi-adjuvant systems (combinatorial admixes) have not been rigorously tested. A more systematic method of studying the effect of different adjuvants on CoV vaccines will be valuable for vaccine development, perhaps using genetic reference populations that more accurately phenocopy human genetic variation (Leist and Baric, 2018).

Trimeric forms of the *S*-protein and RBD have been developed using the T4 trimerization domain to mimic the native conformation of the spike RBD (Kam et al., 2007; Tai et al., 2016). The trimeric RBD antigens are able to elicit a robust nAb response and protect 80% of hDPP4 transgenic mice from lethal MERS-CoV challenge, although most animals still experienced slight weight loss (Tai et al., 2016). Alternatively, the MERS-CoV *S*-protein has been structurally designed to remain in perfusion state by mutating V1060 and L1061 at the tip of the central helix to proline (S-2P) (Kirchdoerfer et al., 2016; Pallesen et al., 2017). The MERS S-2P protein retains the receptor binding properties of the wild-type S and elicits nAbs against at least 3 different S domains, including RBD, NTD, and S2. Intramuscular injection of the MERS S-2P elicits nAb responses in mice comparable to the monomeric S1 and trimeric *S*-protein antigens. (Pallesen et al., 2017).

Similar to subunit vaccines are the viral like particle (VLP) and nanoparticle vaccines. VLP and nanoparticles provide multivalent binding similar to actual viruses without the potential safety concerns. In SARS-CoV, multiple systems were used to generate *S*-protein VLPs, including the mouse hepatitis virus (MHV) and influenza matrix 1 (M1). In the chimeric MHV system, the SARS-CoV protein is co-expressed with the MHV E, M and N proteins to produce MHV-S VLP. Mice vaccinated with the MHV-S VLP and alum have inhibited viral replication in lung after a homologous strain challenge (Lokugamage et al., 2008). Instead of using MHV structural proteins, the influenza system express the SARS-CoV *S*-protein with influenza virus M1 proteins in Sf9 cells to create the M1-S VLP. Immunization of M1-S VLP with aluminum hydroxide can protect mice from a lethal challenge of SARS-CoV (Liu et al., 2011). In MERS-CoV, expression of S, E, and M proteins using the baculovirus system produces VLPs that are morphologically similar to the actual virus (Coleman et al., 2014; Wang et al., 2017b). Other methods such as CPV-based (Wang et al., 2017a), rabies virus (MV)-based (Wirblich et al., 2017), ferritin-based (Seong, 2018), and *S*-protein aggregates (Coleman et al., 2014) are all able to elicit immune responses and reduce viral replication in a mouse model when co-administered with Matrix M1 adjuvant (Coleman et al., 2017). Although all show different degree of immune response in animal, only one study showed a reduction of viral titer *in vivo* via an adenovirus transduced hCD26/DPP4 mouse model (Coleman et al., 2017).

### Clinical Trials for SARS- and MERS-CoV Vaccines

A finished phase 1 clinical trial for a SARS-CoV vaccine is a DNA vaccine that encodes the ectodomain of the SARS-CoV *S*-protein (NCT00099463). DNA vaccines rely on a continuous expression of antigen from a DNA plasmid that is injected intramuscularly and electroporated (Muthumani et al., 2015; Wang et al., 2015; Chi et al., 2017). In pre-clinical studies, 3 doses of an intramuscular plasmid injection was able to reduce viral titer in both lung and nasal turbinate in a BALB/c challenge model (Yang et al., 2004). The phase 1 trial showed favorable results; after 3 doses of DNA vaccine, all subjects showed CD4+ T cell responses, while 80% of subjects had nAbs and 20% of subjects showed CD8+ T cell responses. However, there has been no follow-up in the vaccine development, likely due to the end of the SARS-CoV outbreak (Martin et al., 2008). One MERS-CoV vaccine that is currently undergoing clinical trials (NCT03721718) is a DNA-based vaccine (Modjarrad et al., 2019). GLS-5300 is a DNA vaccine based on a consensus full-length *S*-protein from MERS-CoV under the control of a CMV promoter. In preclinical studies, the vaccine was electroporated into mice, camel and rhesus monkeys three times within 1 month. The vaccine elicited B cell responses in all animals at 1 month post vaccination, and extracted IgG was able to neutralize multiple strains of MERS-CoV including England/2/2013, and Al-Hasa/1/2013 and, surprisingly, a group 1b CoV NL63 and a group 2a CoV HKU1 using the pseudotype neutralization assay (Muthumani et al., 2015). T cell responses were assessed in mice and monkeys, with both demonstrating T cell responses as indicated by the presence of IFN-γ, TNFα and IL2-secreting CD4+ and CD8+ T cells after peptide stimulation. Rhesus monkeys were also protected from challenge of the vaccine strain with lower viral titers and lung pathology as assessed by radiography and pathology studies (Muthumani et al., 2015). Currently, GLS-5300 has completed Phase I clinical trials (safety). Three doses (0.67, 2, and 6 mg) of GLS-5300 were electroporated intramuscularly at weeks 0, 4, and 12. Ninety-four percent of subjects were seroconverted and nAbs were detected in 50% of the individuals. Seventy-six percent of subjects developed T-cell responses against peptides derived from MERS-CoV *S*-proteins (Modjarrad et al., 2019). Other than a full *S*-protein DNA vaccine, different designs also show promising results in preclinical mouse models. Notably, a DNA vaccine composed of only the S1 domain showed efficacy when paired with different adjuvants (Chi et al., 2017). Hybrid strategies using a DNA vaccine paired with a protein booster also showed promising results in eliciting more balanced Th1 and Th2 responses (Wang et al., 2015; Al-Amri et al., 2017).

### Vector-Based Vaccines

Two out of the three clinical trials for MERS vaccines are vectorized vaccines. Viral vector-based vaccines have multiple advantages over the generic protein or DNA-based subunit vaccines. Firstly, as viral vectors utilize a defined viral entry mechanism, they are more efficient at delivering DNA into cells. Second, the vector itself can serve as an adjuvant which in turn elicits both B- and T-cell responses (Ura et al., 2014). Finally, a wide variety of vector systems including measles viruses (Malczyk et al., 2015; Bodmer et al., 2018), Venezuelan equine encephalitis virus (VEEV) replicon system (Deming et al., 2006; Agnihothram et al., 2014; Zhao et al., 2016), adeno-associated virus (AAV) (Du et al., 2008), parainfluenza type 3 (BHPIV3) (Buchholz et al., 2004; Bukreyev et al., 2004), human and chimpanzee adenovirus (hAd5 and ChAdOx1) (See et al., 2006, 2008; Kobinger et al., 2007; Kim et al., 2014; Guo et al., 2015; Hashem et al., 2019) and modified vaccinia virus Ankara (MVA) (Bisht et al., 2004; Czub et al., 2005; Kapadia et al., 2005; Haagmans et al., 2016) have been previously established for use as vaccine platforms for multiple infectious diseases. Herein, we will focus on the three systems that have been or are currently under clinical trial; all others are summarized in Table 1.

Replication-defective adenovirus vectors are one of the most effective choices to deliver vaccine antigens. Human adenovirus 5 (hAd5) and enteric adenovirus type 41 (Ad41) have both been used to deliver MERS-CoV S or S1 proteins. Intramuscular inoculation of the vaccine elicits both B-cell (nAb titer) and T-cell (IFN-γ secreting splenocytes and pulmonary lymphocytes) responses (Guo et al., 2015). However, pre-existing nAbs against hAd5 and 41 in the human population have limited their usage to dromedary camels instead of humans (Chirmule et al., 1999). The pre-existing nAb problem against hAd5 can be circumvented by using a chimpanzee adenovirus. One such platform is ChAdOx1, wherein the adenovirus E1 gene is replaced by a MERS-CoV *S*-protein with an N-terminal secretion peptide from human plasminogen activator (tPA) driven by a CMV promoter. Intramuscular inoculation of the vector successfully elicits nAbs against *S*-protein as quickly as 14 days post infection. Splenic CD8+ T-cells secreting IFN-γ, TNF-α and IL-17 are also present at 28 days post infection (Alharbi et al., 2017). A single intranasal or intramuscular inoculation of ChAdOx1-MERS is able to protect a human DPP4 transgenic mouse from lethal challenge by MERS-CoV, and the vaccine (Munster et al., 2017) is currently under phase 1 clinical trial (NCT03399578). CHAdOx1 has also shown effective protection for Rift Valley Fever Virus in dromedary camels (Warimwe et al., 2016).

The modified vaccinia virus Ankara (MVA) vector is another effective platform for MERS-CoV vaccine development. *S*-protein is inserted into the MAV genome at deletion site III driven by the viral P11 promoter. After a single intramuscular injection, BALB/c mice produce nAbs against both the RBD and S2, as tested *in vitro* (Song et al., 2013). A follow-up study identified IFN-γ secreting splenocytes after peptide S291 stimulation at 56 days post infection, suggesting the vaccine is able to elicit memory CD8+ T cell responses. The vaccine is also able to protect a hDDP4-transduced BALB/c mouse model. RNA genomes in the lungs and lung pathology are drastically reduced compared to mock vaccination (Volz et al., 2015). The MAV based MERS-CoV vaccine has also been tested on dromedary camels and elicits induction of nAbs in sera and nasal swabs. Vaccinated camels also show reduced RNA genomes and gross pathology. The Phase I clinical trial has just been completed and the results are pending (NCT03615911).

## Antivirals

### The Challenge for Treatment Windows

The average incubation period for SARS-CoV and MERS-CoV is around 5 days (Zumla et al., 2015; de Wit et al., 2016) and the main site of viral replication is the lower respiratory tract (Corman et al., 2015; Petrovsky, 2016). At 7–10 days after symptomatic onset, viral RNA titer peaks in the upper respiratory tract (Drosten et al., 2004; Corman et al., 2015). For terminal cases, disease lasts for an average of 12 days post symptomatic onset for MERS-CoV and 24 days for SARS-CoV (Zumla et al., 2015). Interestingly, severe symptoms begin as the viral titer is decreasing, suggesting that severe CoV pathogenesis is due to immune complications and the inability to resolve inflammation (Peiris et al., 2003a; Wang et al., 2004). SARS-CoV upregulates pro-inflammatory cytokine production in the lung, including IL1, IL6, IL8, IL10, CXCL10 and TNF-α production (Binnie et al., 2014). Compared to SARS-CoV infected patients with mild diseases, patients with ARDS fail to induce interferon (IFN) expression and the subsequent IFN-stimulated genes (ISGs) that are indicative of adaptive immune responses (Cameron et al., 2007; Binnie et al., 2014). The inability to switch from an innate immune response to the adaptive immune response may lead to uncontrollable inflammation and severe end stage lung disease. Given the rapid progression of symptoms to terminal illness, there is only approximately a 1 week treatment window after the onset of symptoms for antiviral and medical intervention (Widagdo et al., 2017). This treatment window could further compromised by delays in virus diagnosis, causing a challenge for timely medical intervention administration when the virus titers and pathological symptom are relatively mild (Corman et al., 2015; Ahmed, 2019). Unlike humans, experimental animal models have a compressed disease course (<7 days), and it is difficult to differentiate between early and late phase of infection. It would be beneficial for clinical studies to separate the early- (<10 days) and late-phase (>10 days) patients to determine differences in patient response between the groups.

### The Challenge for Therapeutic Development

Despite of the presence of a 3′ to 5′ exoribonuclease (exoN) proofreading enzyme, their large genome size (28–30 kb) means that CoVs remain in the category of highly mutating viruses (Eckerle et al., 2007, 2010; Denison et al., 2011). The high mutation rate poses a considerable challenge for antiviral development as drug resistant viruses could arise or already exist within the quasispecies in nature or from an infected individual (Briese et al., 2014). For instance, after a prolonged period of nAb or drug treatments, the CoV can acquire mutations which confer resistance to the therapeutics (Zumla et al., 2016a). The appearance of these “escape viruses” has been confirmed in multiple mouse model studies in well-contained laboratory settings as well as in nature (Neuman et al., 2006; Ter Meulen et al., 2006; Huang et al., 2008; Jiang et al., 2014; Tang et al., 2014; Tai et al., 2017; Kleine-Weber et al., 2019). Fortunately, some of the mutations that confer drug resistance also compromise viral fitness and attenuate the virus (Deng et al., 2014; Agostini et al., 2018). Another therapeutic intervention is the use of immune modulators, such as IFN-α2a/2b, IFN-β1b and corticosteroids in treating CoVs (Loutfy et al., 2003; Peiris et al., 2003b; Sung et al., 2004). Although multiple studies have shown efficacy of IFN-α2a/2b and IFN-β1b singly or in combination with off-labeled antivirals such as ribavirin, lopinavir (LPV) and ritonavir (RTV) in treating SARS- and MERS-CoV in mouse, rhesus monkey and marmoset models (Haagmans et al., 2004; Barnard et al., 2006b; Falzarano et al., 2013b; Chan et al., 2015), clinical studies have shown inconclusive results (Momattin et al., 2013; Mo and Fisher, 2016). Currently, a randomized controlled trial is underway to determine the efficacy of a combination of LPV/RTV and IFN-β1b in treating MERS-CoV infection (NCT02845843).

### Convalescent Plasma and Monoclonal Antibodies

**FIGURE 2:**
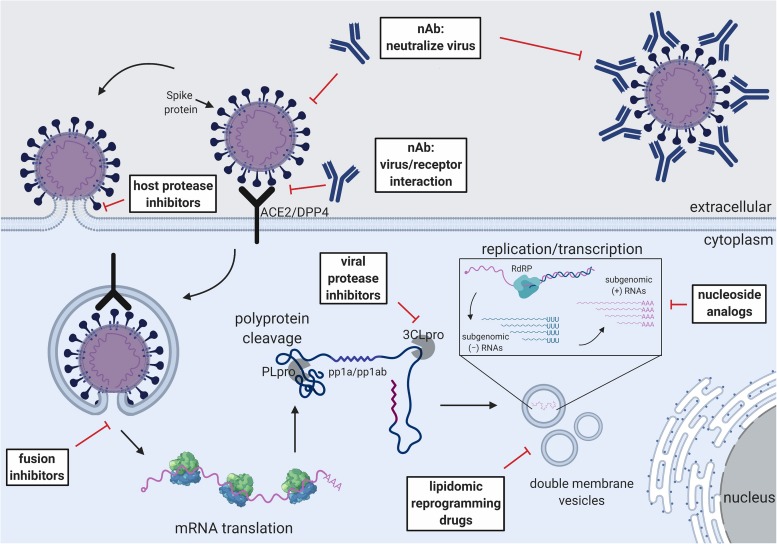
Schematic of the CoV replication cycle and key steps for antiviral targets. White text boxes indicate the subtype of antivirals that work either extracellularly or intracellularly. Different steps of the CoV replication cycle are illustrated in cartoon form, including receptor binding, membrane fusion, viral RNA replication, sub-genomic RNA transcription and translation.

Although there is no approved drug to treat severe CoV infection, multiple strategies have shown promising results in an experimental setting (Figure 2). Convalescent plasma (CP) is derived from patients who have recovered from SARS-CoV and MERS-CoV infection and contains high titer nAbs (Hsueh et al., 2004; Al Kahlout et al., 2019; Shin et al., 2019). Some data suggest that CP use against SARS-CoV infection is safe and, when administered at an early time point, may reduce mortality (Mair-Jenkins et al., 2015). CP neutralizing titers > 1:80 may have a positive impact on infected MERS-CoV patients with respiratory failure (Ko et al., 2018). Unfortunately, CP from convalescent patients is difficult to obtain in large quantities and the Ab titer is too low to have beneficial effects, making it difficult to be used as a main stream therapeutic or for clinical testing (Arabi et al., 2016). As such, no systematic, well-designed clinical trial has formally demonstrated the efficacy of CP in emerging CoV infection. Rather, a clinical trial for testing CP against MERS-CoV infection was withdrawn prior to patient enrollment (NCT02190799).

Although CP from convalescent patients is difficult to obtain, the active component (neutralizing antibodies) can be isolated and subsequently produced in large quantity using recombinant technology (Corti et al., 2016). Passive infusion of neutralizing monoclonal Abs (mAbs) has been used for several diseases with success, including RSV and Ebola virus (Graham and Ambrosino, 2015; Zumla et al., 2016b). Numerous highly potent mAbs against SARS-CoV and MERS-CoV have been isolated by multiple groups all over the world using different methods such as phage display and direct B cell cloning from immunized animals or convalescent patients (Sui et al., 2004; Traggiai et al., 2004; Greenough et al., 2005; van den Brink et al., 2005; Zhu et al., 2007; Corti et al., 2016). While all of them shows protecting activity *in vitro*, several of them have also shown efficacy in mouse and NHP models (Ter Meulen et al., 2004; Johnson et al., 2016; Chen et al., 2017; van Doremalen et al., 2017; de Wit et al., 2018; Xu et al., 2019). The plethora of potent nAbs have also provided insight into the major antigenic sites on which vaccine development should focus. A list of mAbs that show efficacy *in vivo* against SARS-CoV and MERS-CoV are summarized in Table 2. Due to the differences in testing conditions, direct comparison of mAb efficacy should be avoided. Multiple comprehensive review articles on the subject can also be found (Prabakaran et al., 2009; Coughlin and Prabhakar, 2012; Xu et al., 2019).

**TABLE 2:** Summary of SAR-CoV and MERS-CoV neutralizating antibodies.

**SARS mAbs**	**Epitopes**	**Origins**	**Animal models**	**Treatment timing, Routes**	**Dose, route of infection, Strains**	**Results**	**References**
S3.1	Spike	EBV transformated B cell from SARS patient	8 weeks old BALB/c	24 h pre-infection, prophoylatic, IP	10^4^ TCID50, IN, Urbani	Reduce viral titer (lung and nasal turbinates)	Traggiai et al., 2004
80R	RBD	Phage display on naive human antibody library	16 weeks old BALB/c	24 h pre-infection, prophoylatic, IP	10^4^ TCID50, IN, Urbani	Reduce viral titer (lung)	Sui et al., 2004
m396	RBD	Naive human antibody library	8 week old BALB/c	24 h pre-infection, prophoylatic, IP	10^5^ TCID50, IN, Urbani, GD03, SZ16	Reduce viral titer (lung)	Zhu et al., 2007
S230.15	RBD	EBV transformated B cell from SARS patient	8 weeks old BALB/c	24 h pre-infection, prophoylatic, IP	10^5^ TCID50, IN, Urbani, GD03, SZ16	Reduce viral titer (lung)	Zhu et al., 2007
			12 months old BALB/c	24 h pre-infection, prophoylatic, IP	10^6^ PFU, IN, Urbani, GZ02, SZ16		Rockx et al., 2008
				Co-administration, IN	10^6^ PFU, IN, GZ02	Not conclusive, reduce titer	
				1, 2, and 3 days post-infection, theraputic	10^6^ PFU, IN, GZ02	Not protective	
CR3014	RBD	Naive human antibody library	Ferret	24 h pre-infection, prophoylatic, IP	10^4^ TCID50, IT, HKU-39849	Reduce viral titer (lung), no lung lesion	van den Brink et al., 2005
				Co-administration	10^3^ and 10^4^ TCID50, IT, HKU-39849	Reduce viral titer (lung), reduce lung lesion	Ter Meulen et al., 2004
			4 – 6 weeks old BALB/c	24 h pre-infection, prophoylatic, IP	10^5^ TCID50, IN, Urbani, GD03, SZ16	Reduce viral titer (lung)	Greenough et al., 2005
m68	Spike (non-RBD)	Transgenic mice with human Ig gene immunized with SARS-CoV Spike	4 – 6 weeks old BALB/c	24 h pre-infection, prophoylatic, IP	10^5^ TCID50, IN, Urbani, GD03, SZ16	Reduce viral titer (lung)	Greenough et al., 2005

**MERS mAbs**	**Epitopes**	**Origins**	**Animal models**	**Treatment timing, Routes**	**Dose, route of infection, Strains**	**Results**	**References**

4C2	RBD	Mice immunized with MERS-CoV RBD	4 – 6 weeks old BALB/c transduced with hDPP4 by Ad5	24 h pre-infection, prophylactic, IV	10^5^ PFU, IN, EMC/2012	Reduce viral titer (lung)	Li et al., 2015
				24 h post-infection, theraputic, IV			
Mersmab1	RBD	Mice immunized with MERS-CoV S1	hDPP4 transgenic mice	24 h post-infection, theraputic, IV	10^4.6^ TCID50, IN, EMC/2012	Reduce viral titer (lung)	Du et al., 2014; Qiu et al., 2016
CDC2-C2	RBD	Single B cell cloning from MERS patients	hDPP4 transgenic mice	24 h pre-infection, prophylactic, IP	10^6^ TCID50, IN, EMC/2012	Reduce viral titer (lung)	Wang et al., 2015, 2018
G2	Spike (non-RBD)	Mice primed with MERS-CoV DNA and S1 protein	hDPP4 transgenic mice	24 h pre-infection, prophylactic, IP	10^6^ TCID50, IN, EMC/2012	Reduce viral titer (lung)	Wang et al., 2015, 2018
G4	S2	Mice primed with MERS-CoV DNA and S1 protein	hDPP4 transgenic mice	24 h pre-infection, prophylactic, IP	10^6^ TCID50, IN, EMC/2012	Reduce viral titer (lung)	Wang et al., 2015, 2018
MCA1	RBD	Phage display on naive human antibody library	2 years old common marmosets	24 h pre-infection, prophylactic, IP	5 × 10^6^ PFU, IT, EMC/2012	Reduce viral titer (lung), weight loss and patholicical scores	Chen et al., 2017
				2 or 12 h post-infection, theraputic, IP			
LCA60	RBD	Single B cell cloning from MERS patients	4 – 6 weeks old BALB/c transduced with hDPP4 by Ad5	24 h pre-infection, prophylactic, IP	10^5^ PFU, IN, EMC/2012 and London1/2012	Reduce viral titer (lung), weight loss and pathology	Corti et al., 2015
				24 h post-infection, theraputic, IP			Corti et al., 2015
			2 – 6 years old common marmosets	24 h pre-infection, prophylactic, IP	5.2 × 10^6^ PFU, IN + IT + ocular + orally, EMC/2012	Moderately protective, reduce weight loss and pathology	de Wit et al., 2019
m336	RBD	Phage display on navie human antibody library	hDPP4 transgenic mice	12h pre-infection, prophylactic, IP	10^4^ TCID50, IN, EMC/2012	reduce viral titer (lung), weight loss and patholicical scores	Ying et al., 2014, 2015
				12h post-infection, theraputic, IP			Agrawal et al., 2016
			5 – 7 months old Australian white rabbit	24 h pre-infection, prophylactic, IV and IN	10^5^ TCID50, IN, EMC/2012	Reduce viral RNA titer and lung inflammation	Houser et al., 2016
			Common marmosets	6 h (IV) and 48 h (SC) post-infection, theraputic	5.2 × 10^6^ PFU, IN + IT + ocular + orally, EMC/2012	Moderately protective, reduce clinical diseases and gross pathology	van Doremalen et al., 2017
NbMS10	RBD	llama immunized with MERS-CoV RBD	hDPP4 transgenic mice	3 days pre-infection, prophylactic, IP	10^5.3^ TCID50, IN, EMC/2012	Reduce weight loss and increase survival	Zhao et al., 2018
				1 or 3 days post-infection, theraputic, IP			Zhao et al., 2018
HCAb-83	RBD	Dromedary camel immunized with MERS-CoV DNA and Spike protein	hDPP4 transgenic mice	6 h pre-infection, prophylactic, IP	10^5^ TCID50, IN, EMC/2012	Reduce viral titer (lung), weight loss and increase survival	Raj et al., 2018

*IV, intravenous; IN, intranasal; IT, intratracheal; SC, subcutaneous; IP, intraperitoneal.*

The binding epitopes of some well-studied mAbs provide valuable information for the neutralization mechanism and clinical implications. The majority of the mAbs target the RBD of the spike protein and prevent viral attachment. For example, mAb 80R is able to protect both *in vitro* and a 16 weeks old mouse model against SARS-CoV Urbani. However, it is unable to protect other strains due to amino acid variations in the RBD (Zhu et al., 2007). On the other hand, S230.15 mimics receptor binding and triggers conformational changes in the SARS *S*-protein, completely protecting young and old mice from SARS-CoV challenge against multiple SARS-CoV, including Urbani, GD03 and SZ16 (Rockx et al., 2008; Walls et al., 2019a). Similar to SARS-CoV, the majority of mAbs targeting MERS-CoV, such as MERS-4, MERS-27 (Jiang et al., 2014), m336 (Ying et al., 2014, 2015) and humanized Ab 4C2 and 2E6 (Li et al., 2015) all target the RBD and prevent the virus from binding to DPP4 with high potency. Interestingly, the mAb LCA60, isolated from a MERS-CoV infected patient, binds to RBD region and confers a broader neutralizing breadth, and is able to neutralize EMC2012 and London1 strains of MERS-CoV (Corti et al., 2015). Non-RBD targeting Abs G2 and G4 recognize the non-RBD region of S1 and S2 of MERS-CoV, respectively, showing cross-reactivity with multiple MERS-CoV variants and can protect hDPP4 transduced mice from challenge (Wang et al., 2015, 2018). Two nanobodies isolated from camelids, NbMS10 and HCAb-83, show potency in hDPP4 transgenic mice by reducing weight loss and increasing survival after challenge. Interestingly, NbMS10 is able to protect mice as a therapeutic treatment 3 days post infection (Raj et al., 2018; Zhao et al., 2018).

While many mAbs show promising properties for clinical use, two mAbs, REGN3048 and REGN3051, have completed phase 1 clinical trials (NCT03301090). These two mAbs were isolated from VelocImmune mice (expressing the variable regions of human Ig heavy and kappa light chain) immunized with MERS *S*-protein. Both mAbs show picomolar binding and inhibition of MERS pseudo particles transduction on Huh7 cells. REGN3048 is able to neutralize seven natural isolates of S variants, and IP injection of the mAb at 1 day before or after challenge reduces MERS-CoV RNA levels and lung pathologies in an hDPP4 transgenic mouse model (Pascal et al., 2015). Given the acute severe phase that is associated with emerging CoV infections, a major hurdle for therapeutic antibodies and drugs is that early administration will likely prove most efficacious in clinical care, as has also been shown with influenza virus, Ebola virus and RSV immunotherapeutics (Olinger et al., 2012; Qiu et al., 2012; Fry et al., 2014; Rezaee et al., 2017).

### Fusion and Viral Protease Inhibitors

Another critical step for CoV life cycle is membrane fusion and the subsequent release of the RNA genome for replication (Millet and Whittaker, 2018). Membrane fusion of CoVs is governed by the S2 domain of the *S*-protein (Pallesen et al., 2017; Tortorici and Veesler, 2019). The S2 stem undergoes a major conformational change at the two heptad repeat regions (HR1 and HR2) to bring the host and viral membrane in close proximity for fusion pore formation (Yuan et al., 2017; Walls et al., 2019b). Multiple peptidomimetic fusion inhibitors that mimic the HR1 and HR2 of either SARS-CoV or MERS-CoV block the formation of the helical core and efficiently inhibit membrane fusion in the micromolar range (Gao et al., 2013; Lu et al., 2014; Xia et al., 2019). A single report has shown treatment of HR2 peptide 5h prior to MERS-CoV challenge in an Ad5-hDPP4 transduction mouse model reduces viral lung titer (Channappanavar et al., 2015). The ability of these drugs to protect in lethal, high titer mouse models has yet to be proven.

CoV nsp3 and nsp5 genes encode the papain-like cysteine protease (PLpro) and 3C-like serine protease (3CLpro), respectively (Perlman and Netland, 2009). PLpro cleaves the polyprotein and separates it into nsp1 to 4 while 3CLpro separates nsp4 to 16 (Ziebuhr et al., 2000; Harcourt et al., 2004). Since polyprotein processing is a critical step for CoV replication and transcription, viral proteases are high priority drug targets. Originally developed as HIV protease inhibitors, LPV and RTV have low micromolar activity against 3CLpro of both SARS-CoV and MERS-CoV *in vitro* (Wu et al., 2004; De Wilde et al., 2014). Testing in SARS-CoV infected patients has shown beneficial outcomes, including lowering the viral load, reducing the onset of ARDS, and lowering mortality rates with LPV and RTV (see nucleoside analogs) treatments (Chu et al., 2004). However, most of the drug studies were performed using retrospective control, sometimes with unbalanced gender ratios, and no treatment has proven efficacious in a randomized control trial (Zumla et al., 2016a). In a marmoset model, LPV/RTV treatment suggested a modest improvement in clinical and pathological outcome as well as reduction of viral load (Chan et al., 2015). However, due to different pathological consequences of treated and untreated group, viral titers were measured at different time points post-infection and all the experiments were performed with a relatively small number (*n* = 3) of single gender (male) animals (Chan et al., 2015). SARS-CoV and MERS-CoV infection has been shown to be heavily biased by age and gender, where elders and males experience more severe complications than females in clinical cases and mouse models (Karlberg et al., 2004; Alghamdi et al., 2014; Channappanavar et al., 2017). A single subject in a case study, an elderly patient, survived a severe MERS-CoV infection using a combination therapy of LPV/RTV, IFN1α, and ribavirin (Kim et al., 2016). An ongoing clinical trial in Saudi Arabia has begun to use a combination of LPV/RTV and IFNβ-1b for laboratory-confirmed MERS-CoV infection (NCT02845843). Given the importance of 3CLpro I viral life cycle, it is an attractive target for novel drug development (Kumar et al., 2017).

### Nucleoside Analogs and RdRp Inhibitors

Ribavirin is a guanosine analog that targets RNA dependent RNA polymerases (RdRp) and has a broad efficacy against RNA viruses (Loustaud-Ratti et al., 2016). The drug inhibits viral RNA synthesis and has shown efficacy against HCV and RSV (De Clercq et al., 2016). Ribavirin inhibits SARS-CoV and MERS-CoV replication at high concentration *in vitro* (Tan et al., 2004; Falzarano et al., 2013a), However, ribavirin enhances viral replication in the mouse lung and prolongs viral persistence in a SARS-CoV mouse model (Barnard et al., 2006a). Although not effective as a monotherapy, ribavirin shows a synergistic effect against SARS-CoV and MERS-CoV when combine with IFN (Momattin et al., 2013). In a rhesus macaque model, MERS-CoV infected monkeys show improvement after treating with ribavirin and IFN-α2b (Falzarano et al., 2013b). Furthermore, ribavirin has been used in clinical settings during SARS-CoV and MERS-CoV outbreaks (Omrani et al., 2014; Khalid et al., 2015; Shalhoub et al., 2015). While some studies report positive outcome after treatment with ribavirin + IFN, others suggest no significant improvement (Dicaro et al., 2004; Khalid et al., 2015). Due to the variation in dosage and time of administration and the lack of control, the efficacy of using ribavirin in patients is inconclusive. Mechanistically, studies show that recombinant CoV with a deleted proof-reading exonucleases N (ExoN) shows higher sensitivity toward ribavirin. These results indirectly suggest that the low activity of ribavirin in CoV could be explained by the presence of a proofreading exonucleases N (ExoN, nsp14) which can excise the drug from the viral mRNA in CoVs (Smith et al., 2013; Ferron et al., 2017).

Two nucleoside analogs, β-D-N4-Hydroxycytidine (NHC) and GS-5734 (remdesivir), have shown high efficacy against CoVs and are less sensitive to ExoN (Sheahan et al., 2017, 2020; Agostini et al., 2018, 2019). NHC is a cytidine analog and has recently been shown to inhibit multiple viruses, including influenza virus, RSV and Ebola virus (Reynard et al., 2015; Urakova et al., 2017; Yoon et al., 2018). It has micro-molar EC50 against both alpha and beta CoVs including SARS-CoV and MERS-CoV (Barnard et al., 2004; Pyrc et al., 2006; Agostini et al., 2019). Currently under clinical development for Ebola viruses, remdesivir is a nucleoside prodrug that is effective against multiple other RNA viruses including Nipah viruses, RSV, and CoVs (Lo et al., 2017; Sheahan et al., 2017). Remdesivir has sub-micromolar inhibition concentrations in a broad range of CoVs including SARS-CoV, MERS-CoV, and hCoV-NL63, as well as pre-pandemic bat-CoVs WIV1 and SHC014 in an *in vitro* human airway epithelial (HAE) model (Sheahan et al., 2017; Agostini et al., 2018). Prophylactic administration (one day pre-infection) of remdesivir can mitigate disease by reducing the viral titer and lung pathology in lethal mouse models challenged with a mouse adapted SARS-CoV MA15. Remdesivir also shows therapeutic activity when administered early at one day post-infection (corresponding to 7–10 days after the onset of symptoms in human infection). However, treatment initiated two days post infection does not improve disease outcomes, although the murine disease model is more compressed than in humans (Sheahan et al., 2017). Importantly, remdesivir shows excellent prophylactic protection. Rhesus macaques are completely protected from MERS-CoV infection as scored by lung pathology and clinical score as well as inhibited viral growth. Therapeutic treatment 12-h post infection shows moderate improvement of clinical outcomes on NHPs (de Wit et al., 2020). The parental nucleoside of remdesivir, GS-441524, has also shown to be effective for treating FIP, a disease caused by the α-CoV FIPV (Murphy et al., 2018; Pedersen et al., 2019). Currently, NHC and remdesivir are the only broadly effective antiviral drugs against all SARS-like, MERS-like, human contemporary, and animal CoVs (Sheahan et al., 2017; Agostini et al., 2018; Murphy et al., 2018; Pedersen et al., 2019). Some antiviral drugs, such as chloroquine and T-705, also show efficacy *in vitro* and are under consideration for the current COVID-19 outbreak (Wang et al., 2020).

## Host Factor Inhibitors and Immuno-Modulators

### Host Protease Inhibitors

Like all class I viral fusion proteins, CoV S glycoproteins require proteolytic cleavage by host proteases for membrane fusion and viral entry (Belouzard et al., 2012; White and Whittaker, 2016; Millet and Whittaker, 2018). Two cleavage events have been characterized in SARS-CoV and MERS-CoV. The first cleavage event separates the head (S1) and the fusion stem (S2) by cutting the S1/S2 junction (Millet and Whittaker, 2015). The second cleavage event occurs at the S2’ site which is usually located immediately upstream of the fusion peptide (Belouzard et al., 2009). Multiple proteases have been shown to be involved in the cleavage events, including cathepsin-L, trypsin-like serine proteases, transmembrane serine proteases (TTSP) and proprotein convertases such as furin (Millet and Whittaker, 2015). Cathepsin-L inhibitors MDL28170 and SSAA09E1 block SARS-CoV pseudotyped particle infection in pre-treated 293T cells (Simmons et al., 2005; Adedeji et al., 2013). While a peptidomimetic furin substrate, decanoyl-RVKR-chloromethylketone, has been shown to inhibit cleavage of MERS *S*-protein and block infection in multiple cell lines including normal human bronchial epithelial cells (NHBE), the *in vivo* potency of this approach is less certain (Gierer et al., 2014; Millet and Whittaker, 2014; Matsuyama et al., 2018).

### Host Receptor Inhibitors

The blocking of receptor interactions is also a target of antiviral development. *N*-(2-aminoethyl)-1 aziridine-ethanamine (NAAE) blocks the interaction of SARS *S*-protein and ACE2 and inhibits S-mediated cell-to-cell fusion at millimolar concentrations (Huentelman et al., 2004). Similarly, an Ab blocking DPP4 can also inhibit MERS-CoV infection on primary bronchial epithelial cells (Raj et al., 2013). Importantly, the *S*-proteins of SARS-CoVs and MERS-CoV interact with their receptors outside of the active sites. Therefore, it would be of interest to develop inhibitors that do not affect the normal function of the host proteins but abolish the interaction of the CoV and receptor. Otherwise, long term inhibition of cellular proteins could have adverse effects on the host. For instance, inhibition of ACE2 may cause hypertension (Danilczyk and Penninger, 2006). Additionally, DPP4 is also responsible for multiple cellular functions including immune homeostasis, stem cell development, metabolism and T-cell regulation, and hence is not an ideal target for MERS-CoV infection (Matteucci and Giampietro, 2016; Ou et al., 2019).

### Other Host Factor Inhibitors

Another attractive target to inhibit CoV infection is the host metabolic pathways essential for CoV life cycles. CoVs replicate and transcribe in membrane-bound vesicles derived from the host’s rough ER (Snijder et al., 2006; Stertz et al., 2007; Reggiori et al., 2010). MERS-CoV infection upregulates the biosynthetic pathways of multiple major lipogenic enzymes, including fatty acid synthase (FAS), acetyl-CoA carboxylase (ACC) and HMG-CoA synthase (HMGCS) (Yuan et al., 2019). AM580, which targets the major lipid biosynthesis transactivator n-SREBPs by interfering with and downregulating global lipid synthesis (Goldstein et al., 2006; Yuan et al., 2019), inhibits the replication of multiple viruses including influenza virus, Zika virus, Enterovirus-A71 and MERS-CoV (Yuan et al., 2019). Remarkably, MERS-CoV viral titer is reduced by 1,000- to 1,000,000-fold in the presence of AM580 in Huh7 cells or a human intestinal organoid model, respectively, and IP injection of AM580 for 3 days protects a human hDPP4 transgenic mouse model from MERS-CoV lethal challenge (Yuan et al., 2019).

### Immune Modulators

#### Corticosteroids

SARS-CoV and MERS-CoV both cause lung inflammation and can progress into severe respiratory syndrome. Lacking direct antiviral effect, corticosteroids are an immune suppressor which is administrated to severe patients to alleviate lung inflammation. However, immune suppression could also facilitate viral replication. Therefore, corticosteroids are often administrated with antiviral or other immune modulators, such as IFN which can activate the immune system. Retrospective and prospective clinical studies have shown mixed observation in treating SARS-CoV and MERS-CoV patients with corticosteroids, IFN and ribavirin; while some showed a positive effect, others showed no difference.

#### IFNs

SARS-CoV and MERS-CoV are able to suppress the induction of IFN synthesis by multiple mechanisms including inhibition of the IFN signal transduction pathways and evading detection by pattern recognition receptors (PRRs) and toll-like receptors (TLRs) (Frieman et al., 2008; Lim et al., 2016; Mubarak et al., 2019). Therefore, external administration of IFN regiments could re-initiate the antiviral immune response in the host. During the SARS-CoV and MERS-CoV outbreaks, IFN-α, IFN-β, and IFN-γ were used in combination with various antiviral drugs, including LPV/RTV, ribavirin, corticosteroids and poly I:C (Zumla et al., 2016a). Although most of the clinical reports are positive, there is no consensus on the efficacy of IFN treatment in CoV infection. In animal model studies, IFN treatment is only effective when administrated at early time point for both SARS-CoV and MERS-CoV (Channappanavar et al., 2016, 2019). Furthermore, many of the clinical studies were confounded by multiple factors, including IFN dosages, combination of different antivirals, and the stage of infection (Mo and Fisher, 2016). For instance, some studies targeted patients in the late stage of infection and show a worse survival rate than average (Al-Tawfiq et al., 2014; Khalid et al., 2014). Based on multiple research reports, IFN-β shows the best efficacy in treating MERS-CoV infection (Chan et al., 2013; Hart et al., 2014; Kim et al., 2016). Currently, an open labeled, well controlled clinical study is aiming to test a set dose of LPV/RTV and IFN-β in treating MERS-CoV. The trial will probably provide insight into treating MERS-CoV infection in human population.

## The Current Sars-CoV-2 Outbreak: a Challenge, an Opportunity

In December 2019, a new CoV outbreak started in Wuhan, China, a megacity with a population of 11 million. With an estimated basic reproduction number (R_0_) of 1.4 – 2.5, SARS-CoV-2 quickly spread to every province in China and began to spread globally by the end of January 2020 (Zhou et al., 2020), and was declared a worldwide pandemic in March 2020 by the World Health Organization. SARS-CoV-2 is capable of human-to-human transmission via either symptomatic or asymptomatic patients (Rothe et al., 2020). The high mutation rate of CoVs and the possibility of super-spreader could potentiate the continuous spreading globally. Originally from bat, the SARS-CoV-2 Is 96% identical to a bat CoV designated RaTG13, isolated from a cave in Yunnan Province, China and belongs to the betacoronavirus 2b family, as does SARS-CoV (Zhou et al., 2020). However, the *S*-proteins between SARS-CoV and 2019 nCoV share only 76–78% sequence similarity, rendering the current experimental vaccines and antivirals unlikely to be fully protective against SARS-CoV-2. As many group 2b SARS-like CoV have pre-epidemic potential, vaccines and countermeasures should be targeted against all of these strains to maximally protect against current and future threats to the global health and economy (Menachery et al., 2015, 2016).

After two CoV outbreaks in the last two decades, the public health officials and clinicians have experience in preventing spread and treating SARS-CoV-2 patients. Prompt communication between governments, quick quarantine procedures, and rapid viral detection assays have helped minimize SARS-CoV-2 cases in other countries thus far. Additionally, the scientific community has developed novel vaccine strategies and experimental antivirals to fight emerging CoV. New vaccines that specifically targeting SARS-CoV-2 are under development. Moderna had started a phase 1 clinical trial on an RNA vaccine (mRNA-1273) that encodes the prefusion stabilized form of SARS-CoV-2 *S*-protein (NCT04283461). Although there are no published studies of a RNA vaccine platform against CoV, the RNA platform has shown efficacy against multiple other viral infectious diseases including influenza, rabies, Flavivirus, and Ebola viruses in experimental animal models (Zhang et al., 2019). Furthermore, pre-clinical data of the Zika virus mRNA vaccine (mRNA-1893) from Moderna has shown protection against Zika virus and abrogated maternal transmission of Zika virus in pregnant mice (Richner et al., 2017; Jagger et al., 2019). Broad-spectrum antivirals which have shown efficacy against multiple CoVs have the potential to treat SARS-CoV-2 (Sheahan et al., 2017, 2020). Given the high number of infected patients and at risk individuals, the current situation provides an opportunity to initiate clinical trials for (a) vaccine formulation designed to protect uninfected people, (b) broad spectrum antivirals to treat infected individuals and (c) formulation of immune modulators to alleviate clinical pathologies. A well-designed clinical trial could not only ameliorate the current situation, but also lay the foundation for future CoV outbreaks. Supported by a recent study on SARS-CoV-2 (Wang et al., 2020) and multiple research studies on SARS and MERS-CoVs (Sheahan et al., 2017, 2020; de Wit et al., 2020) and a single clinical report (Holshue et al., 2020), remdesivir hold promises as treatment for SARS-CoV-2. The Chinese government has started a clinical trial using remdesivir for treating SARS-CoV-2 patients with mild to severe symptoms (NCT04252664, NCT04257656). On the clinical side, if time and resources permit, a centralized repository that records and digitizes infection cases would aid future medical and epidemiology studies through machine learning programs. Finally, the current situation also provides an opportunity to develop unconventional treatments, such as gene therapy, to target infectious diseases.

## Unconventional Vaccines and Therapeutics – Gene Therapies

The field of gene therapy has undergone rapid growth in the last 10 years. Although mainly focused on rare genetic diseases, its potential in treating infectious diseases should not be discounted. One of the leading vectors, adeno-associated virus (AAV), has proven to be safe for human use and multiple AAV-based gene therapy drugs are approved by the FDA and the EMA (Kay et al., 2000; Kaplitt et al., 2007). So far, the only report of use of AAV in CoV is as a DNA vaccine to deliver SARS-CoV spike protein for immunization (Du et al., 2008). Given the recent developments in human antibody cloning technologies, AAV holds a promising potential to be a hybrid of vaccine and therapeutic which acts as a passive immunization vector to provide protection for the outbreak and as a therapeutic in early time scales.

### AAV as a Vector for Passive Immunization Against Emerging CoV

#### The Vectors – Safety

AAV is a non-pathogenic, non-enveloped, 4.7 kb single-stranded DNA virus belonging to the *Dependoparvovirus* genus within the family *Parvoviridae* (Cotmore et al., 2014). AAV infects a wide variety of animals, from bearded dragons to humans. Natural AAV isolates have different tissue tropisms in humans and can be reverse engineered to better fulfill various medical needs. To target SARS-CoV, MERS-CoV and other respiratory virus infections, a human airway tropic AAV is needed. Multiple reports have demonstrated that natural isolates AAV5 (Zabner et al., 2000), AAV6 (Limberis et al., 2009), and AAV9 (Adam et al., 2014) as well as engineered vectors AAV2.5 (Li et al., 2009) and AAV2.5T (Excoffon et al., 2009) are able to transduce human lung epithelial cells, including primary human airway epithelial (HAE) cultures. Although there is a high prevalence of nAbs against AAVs in the human population, new technologies have been developed to engineer AAV to evade humoral immune responses (Tse et al., 2015, 2017). These developments potentially allow for the delivery of CoV vaccines or immunotherapeutic directly to the mucosal compartments of the lung.

#### The Package – Flexibility

Passive immunization of AAV can be developed as a platform technology in which the nAb can be quickly exchanged to target specific pathogens. Multiple studies have shown passive immunization using AAV is effective against viral infectious diseases such as HIV, (Balazs et al., 2012; Lin and Balazs, 2018) Ebola, (Limberis et al., 2016) influenza, (Balazs et al., 2013; Laursen et al., 2018), and others (Nieto and Salvetti, 2014). The package for delivery is extremely flexible, from authentic immunoglobulins (IgG) to immunoadhesins (IA) to single chain variable fragments (scFv) to bi-specific antibodies (Naso et al., 2017). Furthermore, a combination of Abs, small antiviral peptides and immuno-modulators can be co-delivered at the same time to achieve multidimensional therapy. Although AAV-based passive immunization has not yet been tested as therapeutic, it could serve as a fast-acting prophylactic alternative to traditional vaccines.

#### The Timing – Quick

The most important aspect to control an outbreak is to reduce the spread by protecting the population from infection. However, there is a lag time between the beginning of an outbreak and the development of an effective vaccine in which the population is completely vulnerable. Prophylactic treatments, such as infusion of Abs and antivirals, could protect individual for a short duration. However, these prophylactic treatments require constant intake to stay effective and are toxic as well as financially impractical for long-term use (Hansel et al., 2010). AAV-based passive immunization could perfectly fill the vacuum by protecting the population before the arrival of a vaccine. Since AAV is a platform technology, anti-viral packages can be swapped and tested quickly, within a month (Strobel et al., 2019). After administration in animals, protection can be achieved in less than a week, faster than any vaccine strategies. In an influenza study, AAV9 delivery of IA via intranasal inoculation protected animals from lethal influenza challenges including H5N1, H1N1 and H1N1 1918 within 3 days of AAV administration (Limberis et al., 2013). Unlike traditional gene therapy in which the transgene lasts for long periods of time, the natural turnover rate of airway epithelia means that the nAb introduction is not permanent, reducing the chance that the host will produce antibodies targeting the therapeutic antibody delivered by AAV (anti-drug antibody responses) (Nieto and Salvetti, 2014). Therefore, AAV-based passive immunization is a quick and excellent option to deploy in an outbreak situation for emerging infectious diseases.

### The Challenges

AAV-based gene therapy has great potential for treating viral infectious diseases. However, there are multiple hurdles for AAV-based gene therapy to achieve its full potential. (1) Low transduction efficiency, (2) pre-existing nAb against AAVs, (3) transgene toxicity and loss of expression, and (4) extremely high price tag (Colella et al., 2018; Kaemmerer, 2018). Fortunately, multiple strategies have been developed to address these hurdles (Tse et al., 2015). For instance, through vector engineering, a new generation of AAV vectors can evade pre-existing nAbs while retaining a good transduction profile in the respiratory system (Li et al., 2009; Tse et al., 2017). For rare genetic diseases, life-long gene expression is important for therapeutic purposes but could cause toxicity. On the contrary, for emerging CoVs, the goal is a short-term protection from the virus. Therefore, AAV can target epithelial cells that have a regular turn-over rate, hence providing short-term protection and preventing transgene toxicity. AAV-based therapies are known for their extremely high prices, sometime up to a million US dollar per treatment. However, the prices are inflated due to the small market size of rare genetic diseases and the cost for drug development. Given the huge market size of infectious diseases, the price for AAV-based therapy for infectious diseases should be more reasonable. Unlike rare genetic diseases and other infectious diseases, an emerging CoV outbreak is uniquely suitable for AAV-based passive immunization as a short-term protection therapy before vaccine deployment.

## Conclusion

The continuous development of vaccines, antivirals, and hopefully gene therapies will provide an arsenal for combating and controlling emerging CoV diseases. For vaccine development, understanding the antigenicity and neutralizing antibody footprints of different CoVs could aid the development of broad-spectrum vaccines and, ultimately, the possibility of a universal CoV vaccine. Vaccine formulation, including dosage and adjuvants, should be tested systemically and, if possible, on animal models that reflects the genetic variation of the human population. A deeper understanding of the basic biology of CoV and host-virus interaction can lead to the discovery of druggable targets. Importantly, we should not overlook the potential of the existing broad-spectrum antivirals and should start clinical trials on these drugs in a timely fashion. Prevention is always the best treatment, and constant viral surveillance of wild animals for potential emerging CoVs is extremely important. Testing the outbreak potential of heterologous SARS- and MERS-like viruses with different spike proteins could better prepare society from the next outbreak. A centralized digital database that collect public health and clinical information of the current outbreak would allow global retrospective studies in the future using machine learning and other big data analysis. On the research side, quick, reliable and easily employed viral testing kits should be developed. Innovative technologies such as gene therapy should be adequately explored for their potential to combat CoVs and act as another line of defense against elusive emerging viral diseases. The current SARS-CoV-2 poses a huge challenge for society; however, given experience with emerging CoVs and global effort, it is hoped that the impact of the outbreak will be minimal.

## Author Contributions

LT wrote the review. RM, RG, and RB reviewed and revised the final version.

## Conflict of Interest

The authors declare that the research was conducted in the absence of any commercial or financial relationships that could be construed as a potential conflict of interest.
